# Dual-Crosslinked Gelatin/Dextran Medical Hydrogels Based on Aldimine Condensation and Photopolymerization

**DOI:** 10.3390/gels11110871

**Published:** 2025-10-31

**Authors:** Xia Ding, Bing Yang, Lei Ni, Guangliang Niu, Xinyi Si, Ning Lu, Zhaosheng Hou

**Affiliations:** 1 School of Intelligence Engineering, Shandong Management University, Jinan 250357, China; 14438120160212@sdmu.edu.cn (X.D.); yangbing@sdmu.edu.cn (B.Y.); nilch@163.com (L.N.); 2 College of Chemistry, Chemical Engineering and Materials Science, Shandong Normal University, Jinan 250014, China; 18766175127@163.com (G.N.); 202410100512@stu.sdnu.edu.cn (X.S.); 202410100131@stu.sdnu.edu.cn (N.L.)

**Keywords:** hydrogel, dual-crosslinking, aldimine condensation, photopolymerization, gelatin, dextran

## Abstract

Hydrogels have attracted considerable attention as biomedical materials owing to their distinctive properties; however, improvements in mechanical strength, biodegradability, and biocompatibility remain essential for advanced clinical applications. This study developed a new dual-crosslinked hydrogel based on gelatin (Gel) and dextran (Dex) via sequential aldimine condensation and photopolymerization. Natural Gel and Dex were functionalized to synthesize methacrylated Gel (GelMA) and oxidized Dex (ODex), respectively. An imine-linked network was initially formed between GelMA and ODex via aldimine condensation, followed by a second crosslinked network generated through blue-light-induced free-radical polymerization of GelMA, yielding dual-crosslinked hydrogels (GMODs). ^1^H NMR and FT–IR analyses confirmed the successful functionalization and formation of dual-crosslinked structure. The dual-crosslinked network enhanced the thermal stability and water-retaining capacity of the freeze-dried hydrogels (DGMODs) while reducing the surface wettability and equilibrium swelling ratio of GMODs. The maximum compressive strength (σₘ) increased with crosslinking density; GMOD−2, with moderate crosslinking density, remained intact under 85% compressive strain and achieved σₘ of 108.0 kPa. The degradation rate of GMODs was tunable by adjusting the crosslinking density, thereby modulating their drug-release behavior. GMOD−3, possessing the highest crosslinking density, exhibited effective drug-sustained release for up to five weeks. Biological evaluations, including cytotoxicity assays, live/dead cell staining, and hemolysis tests, verified excellent cytocompatibility (cell survival rate > 92%) and minimal hemolysis ratio (<5%). Furthermore, inhibition zone tests preliminarily revealed moderate antibacterial activity for GMOD−1. The GMOD hydrogels exhibited superior compressive robustness, adjustable biodegradability, and excellent biocompatibility, holding great potential for biomedical applications such as sustained drug-delivery system.

## 1. Introduction

Hydrogels are soft materials consisting of a three-dimensional (3D) porous network structure of hydrophilic polymer molecular chains with water or aqueous phase serving as the dispersion medium. They exhibit remarkable water absorption and retention capabilities [1,2]. Owing to their biomimetic extracellular matrix-like structure and tunable stimulus-responsive behavior, hydrogels show significant potential in biomedical application [3,4,5], such as tissue engineering, regenerative medicine, and drug delivery [6,7,8,9]. However, hydrogels based on synthetic monomers, typically prepared via free-radical polymerization and chemical crosslinking, display superior mechanical properties but suffer from poor degradability. In contrast, those derived from natural polymers offer advantages including biodegradability and high biocompatibility, yet often lack sufficient mechanical strength, which limits their practical application [10,11]. Consequently, there is an urgent need to develop high-strength, biodegradable hydrogels through simple and mild fabrication methods [12,13]. At present, researchers place greater focus on exploring functional hydrogels derived from renewable natural polymers, aiming to meet mechanical performance requirements while maintaining biodegradability and bioresorbability.

Among various natural polymers, collagen [14,15], hyaluronic acid [16], dextran [17], and chitosan [18,19] have garnered significant attention. The biomedical hydrogels based on natural polymers are typically categorized into polysaccharide-based and protein-based hydrogels [20]. Polysaccharides impart hydrogels with enhanced mechanical stability and biocompatibility by forming robust 3D networks, whereas proteins endow them with improved bioactivity and functionality through specific biological motifs.

Dextran (Dex), a biocompatible polysaccharide, has a long history of use as a food additive and as a material in biomedical engineering [21,22]. However, its application in hydrogels is hampered by poor gelation, which necessitates inefficient chemical crosslinking under stringent conditions. Dex is readily oxidized to generate aldehyde-rich oxidized dextran (ODex), which can rapidly form hydrogels under mild conditions via aldimine condensation with amine-bearing polymers [23]. Gelatin (Gel) is a zwitterionic protein obtained from the hydrolysis of collagen. It is valued for its exceptional cell adhesion, biocompatibility, biodegradability, non-immunogenicity, non-toxicity, and cost-effectiveness, making it particularly suitable for diverse biomedical applications, such as tissue engineering and wound healing [24].

Dual-crosslinking architectures offer a distinct advantage over single-network systems, which enables the hydrogels to achieve a balance between strength, flexibility, and resilience, thereby enhancing their load-bearing capacity and resistance to fatigue [25]. Dual-crosslinked hydrogels can be predominantly categorized into three distinct systems: covalent/covalent, covalent/non-covalent, and non-covalent/non-covalent networks [26]. Among these, covalent/covalent dual-crosslinked hydrogels exhibit superior mechanical strength and biostability. However, their fabrication often involves complex procedures and may generate residual cytotoxic byproducts [27]. In contrast, non-covalent dual-crosslinked hydrogels can be readily formed under mild conditions and possess intrinsic self-healing capabilities, though they generally suffered from poor biostability and mechanical robustness [28]. More recently, photo-induced crosslinked hydrogels, which undergo rapid in situ gelation upon light irradiation in the presence of photoinitiators, have attracted considerable interest. These systems integrate several advantageous features, including improved mechanical strength, absence of toxic byproducts, mild crosslinking conditions, and tunable gelation kinetics [29,30], making them highly promising for a broad range of biomedical applications such as tissue engineering, wound healing, and controlled drug delivery.

This work developed a new class of covalent dual-network hydrogels based on Gel/Dex via aldimine condensation and photopolymerization. The fabricated hydrogels were predicted to have enhanced mechanical properties, excellent biocompatibility, and controllable degradability, and to hold promise for a wider range of biomedical applications. Natural Gel and Dex were chemically functionalized to synthesize methacrylated Gel (GelMA) and aldehyde-rich ODex, respectively. The primary imine-linked network formed via aldimine condensation between GelMA and ODex, followed by the formation of secondary network through blue-light-induced free-radical polymerization of GelMA, producing dual-crosslinked hydrogels (GMODs). The influence of crosslinking density on microstructure, thermal properties, surface hydrophilicity, water retention, swelling capacity, compressive performance, biodegradability, and drug-release behavior of the hydrogels (or freeze-dried hydrogels) was investigated comprehensively. Preliminary biological assessments, including cytotoxicity and hemolysis, were conducted to evaluate the biosafety of the developed hydrogels.

## 2. Results and Discussion

### 2.1. Formation of GMOD Hydrogels

The sol–gel phase transition process was assessed using the test tube inversion method [31], and the hydrogel was considered to be formed when no flow was observed upon inverting the tube within 10 s. The typical gelation process of GMOD−2 is shown in Figure S1. At the initial stage, the GelMA/ODex mixture exhibited low viscosity and behaved as a free-flowing liquid (Figure S1a). As aldimine condensation occurred between the –NH_2_ groups of GelMA and the –CHO groups of ODex, the first imine-linking network was formed. The viscosity significantly increased within 30 s, but the solution still flowed slowly upon tilting the vial (Figure S1b). The subsequent irradiation with blue light (λ = 405 nm) induced rapid photopolymerization of the vinyl groups in GelMA, forming a secondary network. The solution became fully solidified with no observable flow upon inversion of the vial (Figure S1c). The gelation time for GMOD−0, −1, −2 and −3 were 10, 8, 6, and 5 s, respectively. This trend was attributed to the increased molar ratio of –CHO to –NH_2_ in the system, which facilitated the formation of a denser imine bond cross-linking network, thereby enabling faster formation of the secondary network under light. To obtain samples with more stable properties, all GMODs were exposed to blue light for 60 s after their initial gel formation. It should be noted that the viscous solution with the first network could be injected into the desired site and then rapidly solidified under blue light irradiation, demonstrating its potential as an injectable hydrogel for irregular osteochondral defects or post-resection cavities. Furthermore, photo-induced curing process could be triggered by external irradiation or by fiber-optic illumination delivered via endoscopic devices.

### 2.2. FT-IR Spectra

**Figure 1:**
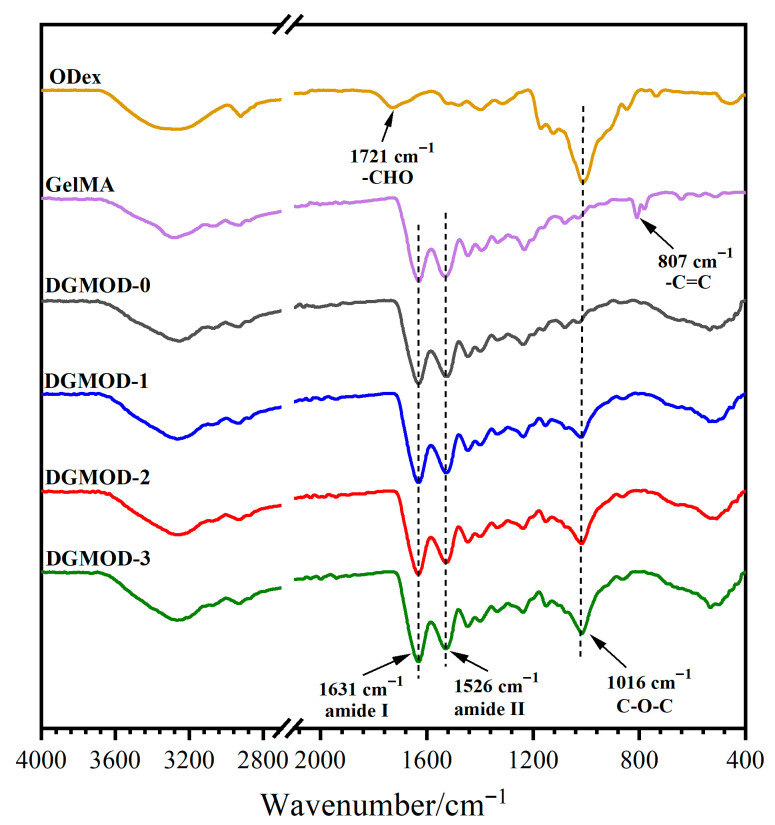
FT−IR spectra of ODex, GelMA and DGMODs.

The FT-IR spectra of ODex, GelMA and DGMODs are displayed in Figure 1. From the spectrum of ODex, it could be found that a stretching vibration band of –CHO appeared at 1721 cm^−1^ [32], indicating the successful oxidation and formation of –CHO groups. The strong absorption peak at 1016 cm^−1^ corresponded to the characteristic vibration of the cyclic C–O–C, which suggested that a large amount of the glucose rings remained unoxidized. In the FT-IR spectrum of GelMA, a bending vibration peak of –C=C was observed at 807 cm^−1^, confirming the successful grafting of vinyl groups onto the Gel molecular chains. The characteristic absorption peaks of –CHO and –C=C completely disappeared in the spectra of DGMODs, which indicated that, under an excess of –NH_2_ groups, –CHO groups had fully participated in the aldimine condensation, while the –C=C groups underwent complete free-radical polymerization under blue-light irradiation. Moreover, the strong absorption bands at 1631 and 1526 cm^−1^ were assigned to the amide I and II of the GelMA component, respectively. The absorption band at 1016 cm^−1^ corresponded to the C–O–C stretching of the ODex glucose rings, and the absorption intensity increased progressively with the ODex content in the DGMODs (DGMOD−0~−3). The characteristic imine (–CH=N–) absorption peak (~1620 cm^−1^) was overlapped with the strong amide I band [33], and thus was not observable in the spectrum.

### 2.3. Microstructure and Porosity

**Figure 2:**
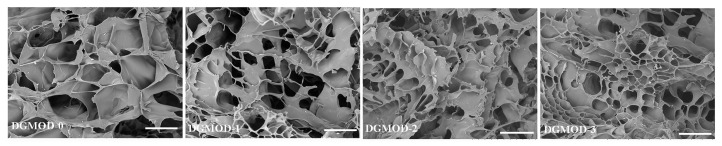
SEM images of DGMODs (scale bar: 500 μm).

The microstructure of a hydrogel is closely related to its mechanical properties. Hydrogels with uniform and dense network structures can effectively dissipate external forces and exhibit superior compressive resistance [34]. The microstructures of DGMODs with various ODex contents were observed by SEM, as presented in Figure 2. All samples displayed homogeneous internal morphologies with porous and honeycomb-like structures. The pore size progressively decreased with increasing ODex content (DGMOD−0~−3). Since the DGMODs had the same solid content (10 wt%), the pore size of the DGMODs was primarily governed by the crosslinking density. DGMOD-0, which underwent only the photopolymerization of C=C bonds, formed a single network with the lowest crosslinking density and exhibited the largest pore size. In contrast, DGMOD−1 to −3 possessed dual interpenetrating networks formed by aldimine condensation and photopolymerization, which were intricately intertwined and substantially enhanced the overall crosslinking density, resulting in a reduction in pore size. As the ODex content increased, the crosslinking density also increased, leading to a higher number of crosslinking sites per unit volume and the formation of denser network structures, thereby decreasing the pore sizes.

The porosity of the DGMODs was determined using a solvent replacement method. The results revealed high porosities for all samples, with DGMOD−0 to −3 exhibiting porosities of 81.7%, 75.5%, 71.4%, and 68.7%, respectively. This trend was consistent with the increasing crosslinking density. Porosity played a crucial role in determining the swelling behavior and compressive properties of hydrogels. A larger porosity allowed more water molecules to penetrate the crosslinked network, resulting in higher water absorption and swelling capacity. Conversely, smaller porosity indicated a denser structure, which facilitated more effective dissipation of external force under compressive deformation, thereby improving the compressive stability of the hydrogel. These relationships demonstrate that the physicochemical properties of the hydrogel can be effectively tailored by modulating the crosslinking density to control porosity. Moreover, the freeze-dried hydrogels were able to reabsorb water and recover their hydrogel state after immersion in PBS, and the regenerated hydrogels largely retained their original properties. This feature offered practical benefits for storage and transportation.

### 2.4. Thermal Performance

**Figure 3:**
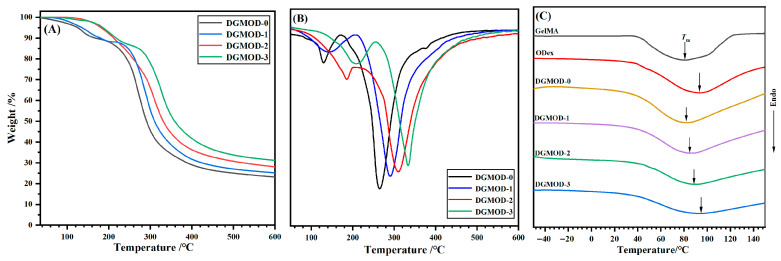
(**A**) TGA, (**B**) DTGA, and (**C**) DSC curves of GelMA, ODex and DGMODs.

TGA technique was used to evaluate the thermal stability of DGMODs. The TGA and DTGA curves are displayed in Figure 3A,B, respectively, and the corresponding parameters are summarized in Table S1. As shown in the TGA curves (Figure 3A), all DGMOD samples exhibited minimal weight loss below 100 °C, which should be attributed to the evaporation of residual moisture in the samples. The temperatures (*T*_5*%*_, ℃) at 5% weight loss (*T*_5%_) for DGMOD−0~−3 were 125.3, 135.2, 181.1 and 186.2 °C, respectively. The gradual increase in *T*_5%_ indicated an improvement in initial thermal stability with increasing ODex content. This enhancement could be ascribed to the higher crosslinking density in DGMOD networks, which strengthened the intermolecular interactions and required more energy to initiate thermal decomposition [35]. The residual weight (*W*_r_) at the end of the test (600 °C) increased progressively, with *W*_r_ values of 23.1%, 25.1%, 28.0%, and 31.2% for DGMOD−0 to −3, respectively. This implied that the thermal stability at the high temperature region also increased with the increasing crosslinking density. As shown in the DTGA curves (Figure 3B), the pyrolysis process of DGMODs occurred in two main stages. The first stage with maximum decomposition temperature (*T*_1_) of 135.3~209.3 °C exhibited a relatively small mass loss, corresponding to the pyrolysis of side-chain groups of Gel molecules. The second stage with *T*_2_ of 264.7~333.6 °C was mainly attributed to the cleavage of peptide bonds in the gelatin backbone and glycosidic linkages in Dex, leading to a major mass loss. Overall, the dual-crosslinked network effectively enhanced the thermal stability of DGMODs, making them suitable for high-temperature sterilization in medical applications.

The DSC curves for DGMODs are presented in Figure 3C, and the corresponding thermal transition parameters are listed in Table S2. No distinct glass transition temperature (*T*_g_) was observed for GelMA, ODex, and DGMODs, which could be attributed to the presence of abundant hydrophilic groups (e.g., –OH) in the materials. These hydrophilic moieties enable a certain amount of residual or absorbed water to remain even after lyophilization, and the retained water acted as a plasticizer that reduced the energy barrier for polymer chain motion, thereby leading to the disappearance or downward shift of *T*_g_ [36,37]. All samples exhibited broad and weak endothermic peaks (*T*_m_) between 40 °C and 120 °C, which corresponded to the melting of crystalline regions. Although the chemical modifications partially disrupted the original hydrogen-bonded structures, the helical segments in Gel chains still retained partial crystallinity, while the partial molecular ordering in ODex was likely attributed to the formation of hydrogen bonds between –CHO and –OH [38]. In the case of DGMODs, the dual-crosslinked networks disrupted the original hydrogen bonds and significantly restricted chain mobility [39], resulting in smaller enthalpy changes (Δ*H*) compared to GelMA and ODex. In addition, as the crosslinking density increased from DGMOD−0 to −3, the Δ*H* values gradually decreased (Table S2). Nevertheless, the Δ*H* values of GelMA, ODex, and DGMODs were all below 21 J·g^−1^, confirming that these materials exhibited an amorphous nature.

### 2.5. Surface Wettability and Swelling

**Figure 4:**
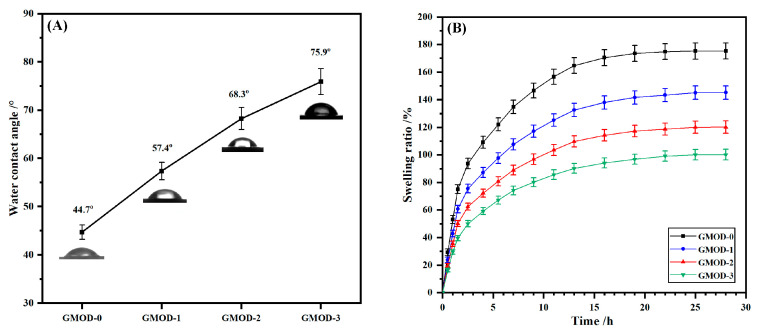
(**A**) Water surface contact angles and (**B**) time-dependent swelling ratio of GMODs (*n* = 5).

The surface wettability of the hydrogels was evaluated by measuring the water contact angle (WCA), as displayed in Figure 4A. The WCA values for GMOD−0 to −3 were 44.7 ± 1.5°, 57.4 ± 1.8°, 68.3 ± 2.3°, and 75.9 ± 2.7°, respectively. The progressive increase in WCA indicated a corresponding reduction in surface wettability. This tendency was mainly attributed to the increased crosslinking density within the hydrogel, which reduced the availability of hydrophilic groups in the Gel and Dex chains. Furthermore, as the dual-network structure became denser, the pore size of the hydrogel matrix decreased, resulting in a more compact surface morphology.

The swelling behavior of hydrogels directly reflects their intrinsic hydrophilicity. Figure 4B illustrates the time-dependent swelling ratio (SR) of GMOD hydrogels at room temperature. The SR values increased rapidly during the initial stage, exceeding 50% within 3 h, and then gradually approached equilibrium after approximately 20 h. Representative optical images of GMOD−2 before and after equilibrium swelling are provided in Figure S2. After equilibrium swelling, the hydrogel expanded significantly with the diameter increasing from 10 mm to 22 mm and transparency enhanced. The equilibrium SR values for GMOD−0~−3 were 175.3 ± 8.8%, 145.1 ± 7.3%, 120.1 ± 6.0%, and 100.1 ± 5.0%, respectively, indicating a notable decrease. This behavior was mainly due to variations in the crosslinking density under constant solid content [40]. A higher crosslinking degree formed a tighter 3D polymer network with smaller pore size, which restricted water absorption and retention, resulting in a lower equilibrium SR. Overall, these results showed that the equilibrium swelling properties of GMOD hydrogels could be effectively adjusted by regulating the crosslinking density at constant solid content.

### 2.6. Water-Retention Behavior

**Figure 5:**
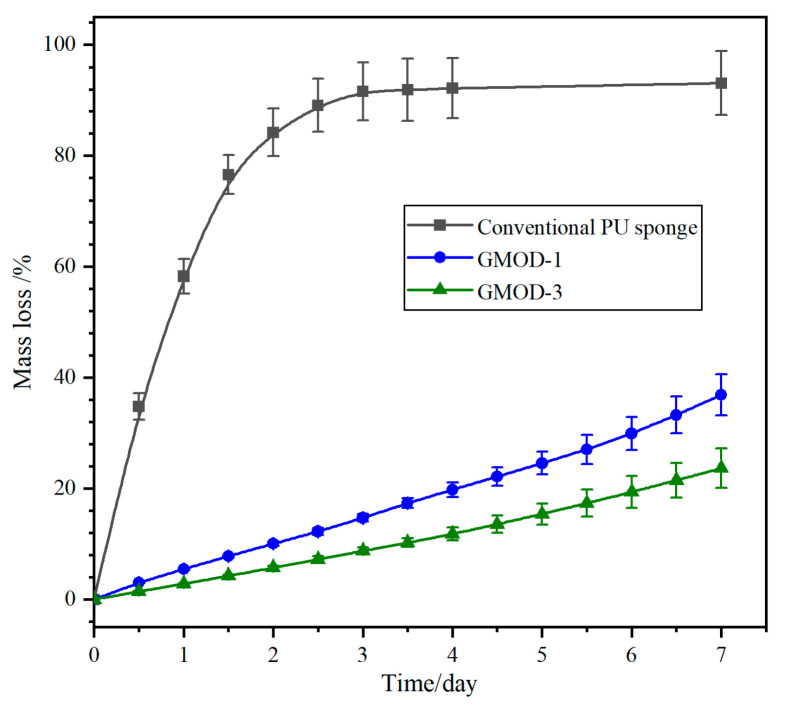
Time-dependent water mass loss of PU sponge, GMOD−1 and GMOD−3 at 25 °C and 30% RH (*n* = 3).

The water-retention capability of hydrogels is a critical factor for their practical applications in medical fields. Figure 5 presents the water loss rate (WLA) profiles of GMOD−1 and −3 with a commercial PU sponge as a comparative sample. Both the PU sponge and the hydrogels lost water over time; however, the PU sponge exhibited a remarkably rapid dehydration process, with a WLA value exceeding 85% within 2 days and nearly complete water loss (>92%) after 4 days. In contrast, GMOD−1 and −3 displayed significantly lower water loss. After 4 days, their water loss was only 19.8% and 11.8%, respectively, and even after 7 days, they retained more than 60% of their initial water content. The water-retention capability of GMODs was comparable to that of Gel-based hydrogels with a double-hydrophilic coating, which retained 72.5% water at 25 °C and 40% RH after 5 days [41]. This enhanced water-retention performance in GMODs should be attributed to the synergistic effect of the dual-crosslinked network and the hydrophilic polymer chains, which more effectively constrained the mobility and evaporation of water molecules. Moreover, due to the higher crosslinking density and smaller pore size, the network structure of GMOD−3 provided stronger confinement on water molecules, thereby endowing it with enhanced water-retention capability. In clinical practice, this pronounced water-retention capacity enabled long-acting dressings for moist wound healing (e.g., corneal protective coatings) and sustained the viability of encapsulated cells/biologics.

### 2.7. Compressive Performance

**Figure 6:**
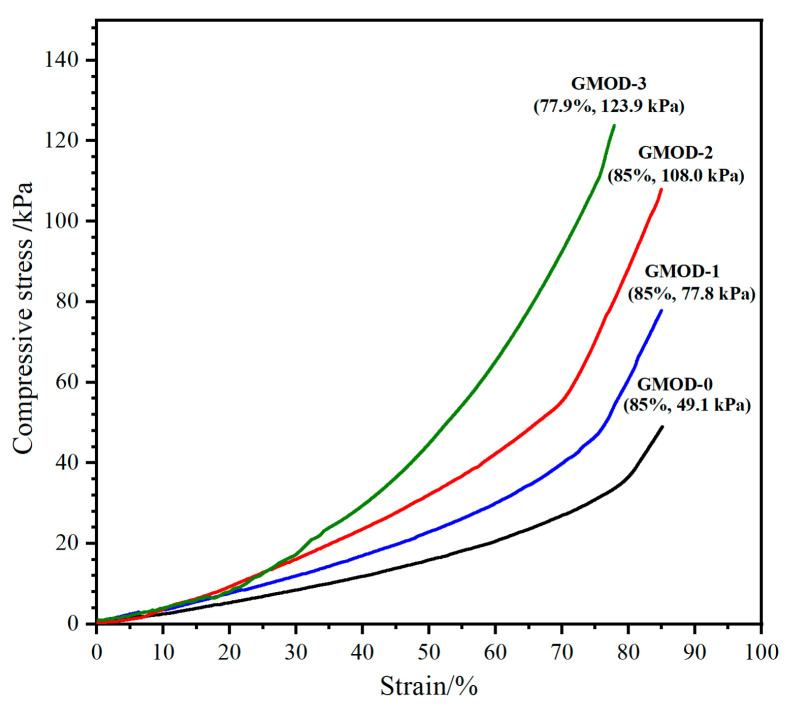
Typical compressive stress–strain curves of GMODs.

The compressive resistance of hydrogels plays a key role in determining their ability to withstand external loads and maintain structural integrity. The compressive performance of GMODs were evaluated through compression tests. To prevent potential damage to the testing instrument, the maximum compressive strain (ε_m_) was limited to 85%. The representative compressive stress–strain curves of GMODs are illustrated in Figure 6. For GMOD−0, which contained only photo-crosslinked network, the maximum compressive strength (σ_m_) was relatively low at 49.1 ± 2.3 kPa. In contrast, the dual-crosslinked GMOD−1, −2, and −3 exhibited markedly enhanced σₘ values of 77.8 ± 3.2 kPa, 108.0 ± 3.8 kPa, and 123.9 ± 4.2 kPa, respectively, demonstrating a progressive increase with crosslinking density. It was evident that the dual-crosslinking strategy and increased crosslinking sites formed a more complex and interconnected network architecture, potentially forming a partially interpenetrating network, allowing the hydrogels to withstand greater compressive load [42]. Although the compressive strength of these dual-crosslinked hydrogels was significantly higher than that of conventional single-network gelatin-based hydrogels [43], it is noteworthy that excessive crosslinking density could increase the brittleness of the hydrogel. This phenomenon was clearly evidenced by the fracture of GMOD−3 at a compressive strain of only 77.9%, confirming the predicted reduction in ductility at high crosslinking densities. Therefore, although enhancing the crosslinking density effectively improved compressive resistance, it should be carefully optimized according to the target requirements of the intended application.

### 2.8. Biodegradability

**Figure 7:**
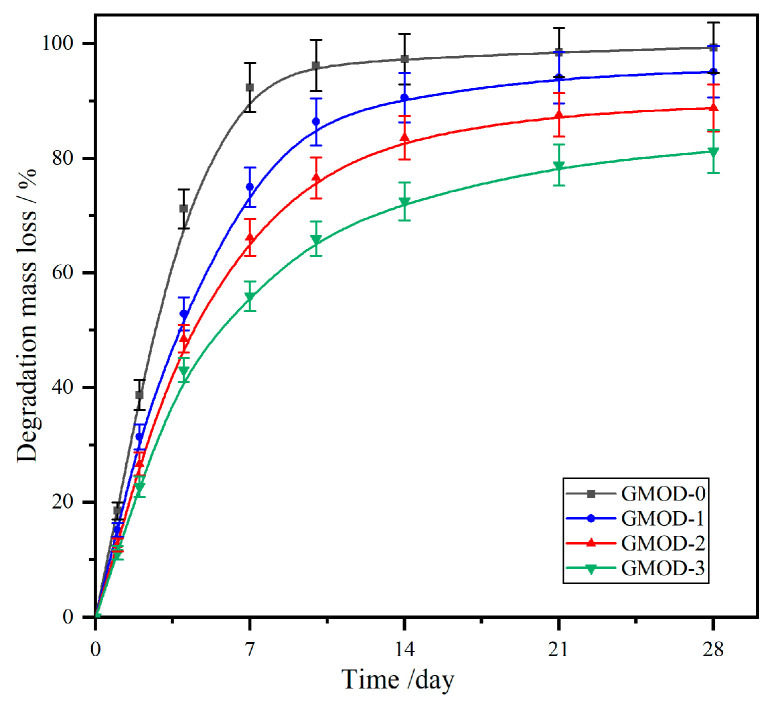
Mass loss curves of GMODs against degradation time in PBS (pH 7.4) at 37 °C (*n* = 3).

The fabricated GMOD hydrogels, primarily composed of biodegradable and bioabsorbable Gel (protein) and Dex (polysaccharide), are expected to be inherently biodegradable. The in vitro degradation behavior of the GMODs was assessed by monitoring time-dependent mass loss in PBS at 37 °C, and the corresponding degradation profiles are presented in Figure 7. All samples displayed a two-stage degradation profile characterized by an initial rapid mass loss followed by a slower phase. With the increase in crosslinking density (GMOD–0~–3), the degradation loss rate (DLR) progressively decreased, resulting in a longer time to achieve near-complete degradation (>95%). Specifically, the GMOD–0 with single crosslinked network exhibited a DLR value of 96.2% after 10 days of degradation, indicating complete degradation. GMOD–1, which possessed the lowest crosslinking density among the dual-crosslinked systems, showed an 86.4% DLR following 10 days of incubation and reached almost complete degradation (95.1%) by the 21st day. In contrast, GMOD–2 and –3, with higher crosslinking densities, displayed considerably slower degradation rate, with DLR values of only 88.8% and 81.2% at the end of the experiment (28 days). These results demonstrated that the degradation behavior of the hydrogels was predominantly governed by their crosslinking density. A denser dual-crosslinked network retarded the diffusion of water, thereby slowing the hydrolytic degradation process [43]. Consequently, the degradation rate of the GMODs could be effectively tailored by adjusting their crosslinking density. Synchronizing hydrogel degradation with tissue healing process facilitated natural tissue ingrowth and regeneration. Moreover, as the hydrogel matrix degraded, it afforded sustained and localized release of encapsulated therapeutics, enhancing treatment efficacy while minimizing side effects.

### 2.9. Drug Sustained-Release Behavior

**Figure 8:**
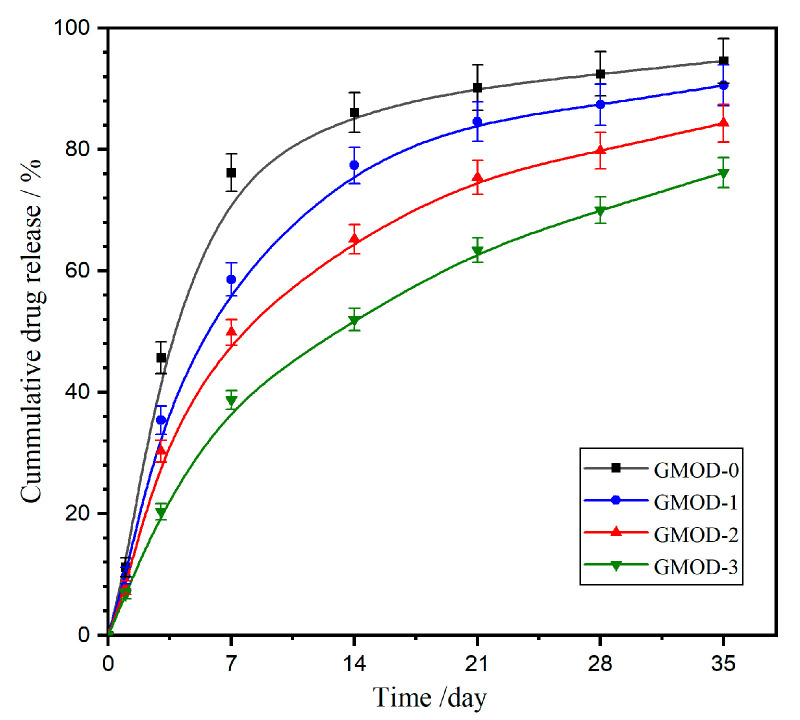
Drug release profiles from GMODs in PBS (pH 7.4) at 37 °C (*n* = 3).

Cefazolin sodium (CEF), a first-generation cephalosporin antibiotic, remains widely used for the treatment of infections in the respiratory, urinary, and gastrointestinal tracts caused by susceptible microorganisms. Figure 8 illustrates the in vitro drug release profile of CEF-loaded GMOD hydrogels in PBS at 37 °C. The drug release process was clearly divided into two distinct stages: an initial burst-release phase followed by a subsequent sustained-release phase. The burst-release stage was primarily governed by a combination of diffusion- and matrix-related factors. First, owing to the high water solubility of CEF, the drug molecules located on or near the hydrogel surface were rapidly desorbed into the surrounding medium. Secondly, the concentration gradient between the hydrogel interior and the external PBS solution generated an osmotic driving force that facilitated drug diffusion. Thirdly, during the initial immersion, the hydrogel network underwent significant swelling, loosening the polymeric matrix and promoting faster molecular transport. Following the initial stage, the release rate gradually decreased, entering a sustained-release phase mainly controlled by the progressive degradation of the hydrogel matrix. The overall CEF-release profile closely mirrored the degradation-induced mass loss trend of GMODs (Figure 7), confirming a degradation-controlled release mechanism. Nevertheless, the cumulative drug release (CDR) values were slightly lower than the corresponding degradation-induced mass loss data. This discrepancy could result from microgel fragments dispersed in PBS that were not quantitatively collected but still retained unreleased CEF molecules. Quantitatively, GMOD−0 exhibited a rapid release pattern, with 76.2% CDR within the first week and nearly complete release (90.2%) after three weeks. A progressive reduction in release rate was observed from GMOD−1 to −3, with CDR values of 58.6%, 49.9%, and 38.7% at one week, and 90.6%, 84.4%, and 76.2% at five weeks, respectively. The inverse relationship between crosslinking density and drug-release rate suggested that higher crosslinking density effectively suppressed hydrogel degradation and drug diffusion, resulting in an extended drug-release duration. Notably, the effective release period of the GMODs was much longer than that of reported Gel/Dex-based hydrogels crosslinked via Diels–Alder click reaction, which exhibited an effective drug release within approximately 6 h [44]. Therefore, the GMOD system demonstrated a substantially prolonged and controllable release behavior, providing a promising platform for sustained drug-delivery applications.

### 2.10. Cytocompatibility

**Figure 9:**
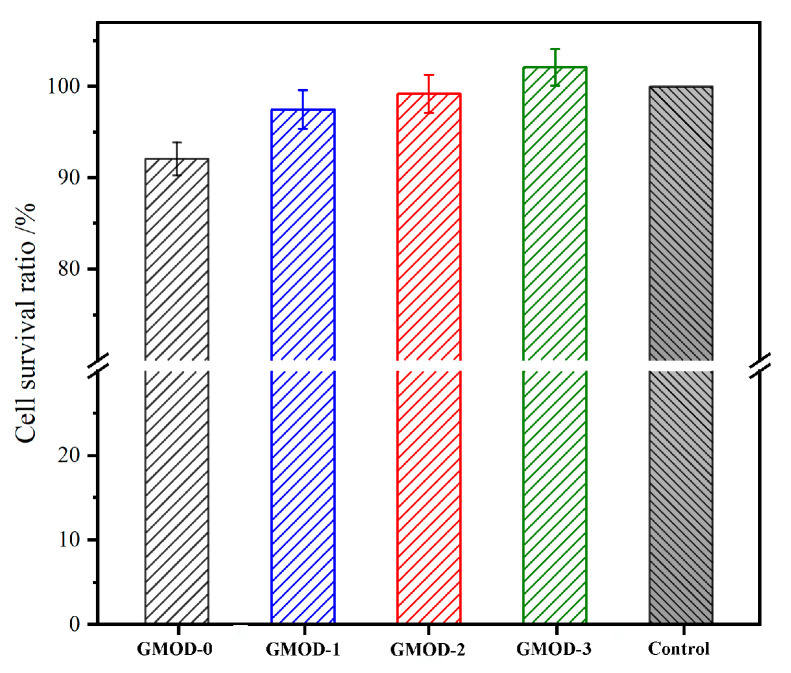
Cell survival rate in GMODs extracts by MTT (37 °C, 72 h, *n* = 3).

**Figure 10:**
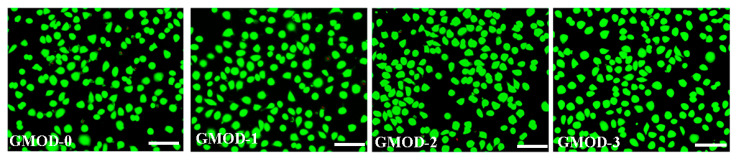
Live/dead cells in GMODs extracts (37 °C, 72 h, scale bar: 200 μm).

Cytocompatibility represents a fundamental prerequisite for evaluating the biocompatibility of biomaterials. In this work, the cytocompatibility of the prepared hydrogels was assessed using cytotoxicity tests and live/dead cell staining with L929 as the model cell. Figure 9 displays the cell survival rates (CSRs) of L929 cells cultured in GMOD extracts for 72 h, as determined by the MTT assay. The CSR values of GMOD−0 to −3 showed a gradually upward trend, with GMOD−3 surpassing the control group and reaching 102.1%. This enhancement in cell viability could be explained by two synergistic effects. First, a higher crosslinking density resulted in a more compact polymeric network, which effectively restricted the diffusion of potentially cytotoxic small molecules into the extract. Second, Dex, one of the main hydrogel components, was a polysaccharide whose degradation products (monosaccharides) could serve as a nutrient source for cell proliferation. Therefore, a higher Dex content in GMODs promoted cellular growth, reflected in elevated CSR values. Overall, all GMOD samples exhibited CSR values exceeding 92%. According to the cytotoxicity classification criteria in GB/T 16886.1-2022 [45], these results corresponded to Grade 1 or 0, indicating negligible cytotoxicity and confirming that the hydrogels satisfied the basic requirements for in vivo biomedical applications [46]. The live/dead staining images for L929 cells cultured in GMOD extracts are exhibited in Figure 10. After 72 h of incubation, most cells emitted green fluorescence and displayed a well-spread morphology, indicating excellent viability, whereas only a few red-stained (dead) cells were detected. These microscopic observations were consistent with the MTT assay results, further confirming that GMOD hydrogels possessed excellent cytocompatibility.

### 2.11. Hemolysis Assay

**Figure 11:**
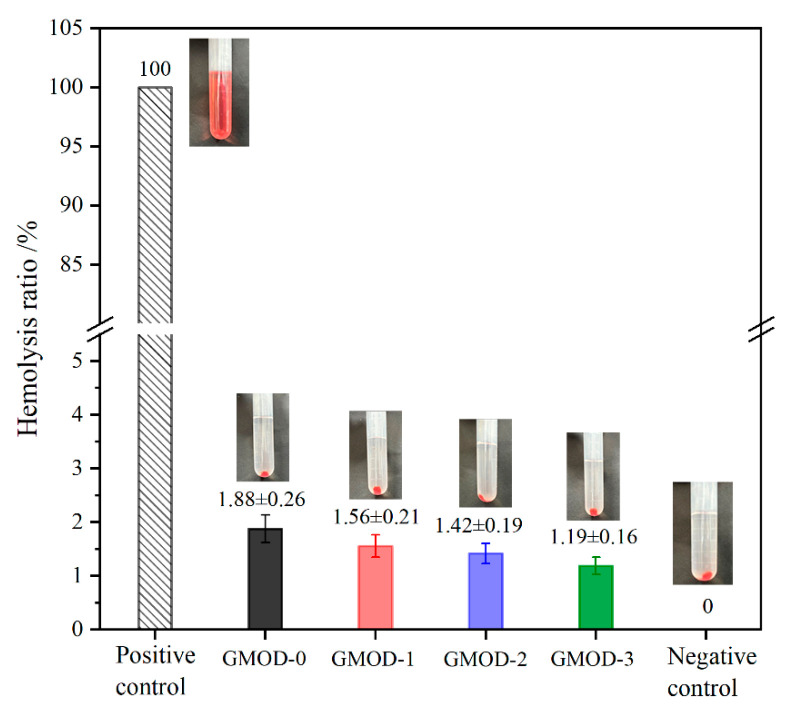
Hemolysis test images and hemolysis rates of GMODs (*n* = 3).

In clinical applications, biomaterials inevitably come into contact with blood. The in vitro hemolysis assay is a simple and effective method to evaluate the hemocompatibility of biomaterials [47]. In this study, anticoagulated fresh rabbit blood was employed to assess the hemolytic behavior of GMOD hydrogels, and the corresponding hemolysis ratios (HRs) and visual results are presented in Figure 11. The positive control (PC, distilled water) group exhibited a distinct red coloration, which was ascribed to the rupture of erythrocytes and the subsequent release of hemoglobin. All GMOD samples displayed clear and transparent supernatants, similar to those of the negative control (NC, normal saline) group, indicating negligible hemolytic activity. When the HR was defined as 100% for the PC and 0% for the NC, the HRs of GMOD−0 to −3 were determined to be 1.88%, 1.56%, 1.42%, and 1.19%, respectively, showing a mild decrease with increasing crosslinking density. All HR values remained well below the 5% threshold, confirming that the prepared hydrogels induced minimal hemolysis and exhibited good hemocompatibility.

### 2.12. Antibacterial Activity

**Figure 12:**
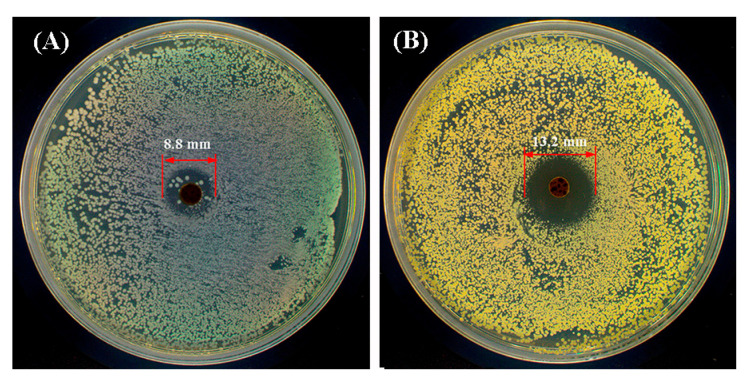
Antibacterial effect of GMOD−1 against (**A**) *E. coli* and (**B**) *S. aureus*.

Bacterial infection during wound healing may lead to persistent inflammation or other complications. Hydrogels with antibacterial activity can effectively prevent infection and therefore facilitate tissue regeneration. Based on the previous study, materials containing −NH_2_ groups possessed intrinsic antibacterial activity [48]. In this work, the GMOD−1, which had the highest −NH_2_ content among the dual-crosslinked hydrogels, was selected for antibacterial testing. The inhibition zone method was used to evaluate its antibacterial activity against *E. coli* and *S. aureus*, and the results are illustrated in Figure 12. For *S. aureus*, a distinct inhibition zone with a diameter of 13.2 mm was observed around the sample, indicating a certain antibacterial effect against Gram-positive (GP) bacteria. In contrast, the inhibition zone formed against *E. coli* measured only 8.8 mm in diameter, and a small number of bacterial colonies were still visible within the zone, suggesting relatively weak inhibitory activity against Gram-negative (GN) bacteria. This difference could be attributed to the structural characteristics of GN bacteria, which possessed a thicker peptidoglycan layer and an additional outer lipid membrane that served as a robust protective barrier against antibacterial agents [49]. The antibacterial activity of GMOD-1 was much lower than that of previously reported chitosan nanoparticles, which exhibited stronger inhibition against both GN and GP bacteria owing to their higher density of -NH_2_ groups [50]. Although GMOD−1 possessed a certain degree of antibacterial activity, further studies are needed to quantitatively assess the impact of −NH_2_ content and to improve the hydrogel’s antibacterial efficacy.

## 3. Conclusions

In this study, a new class of Gel/Dex-based medical hydrogels (GMODs) was developed through a dual covalent crosslinking strategy combining aldimine condensation and photoinitiated polymerization. The resulting dual-network architecture significantly improved thermal stability, water-retention capacity, and compressive performance, while reducing surface wettability and equilibrium swelling ratio. GMODs exhibited tunable biodegradability, and their drug release behavior was mainly governed by the degradation rate, with GMOD−3 achieving a sustained release duration up to five weeks. Biological evaluations confirmed excellent cytocompatibility (CSR > 92%) and minimal hemolytic activity (HR < 5%). In addition, preliminary antibacterial tests revealed that GMOD−1 possessed a certain degree of antibacterial activity. The fabricated GMOD hydrogels, characterized by compressive robustness, adjustable degradability, and excellent biocompatibility, demonstrated great potential for biomedical applications such as controlled drug-delivery systems.

## 4. Materials and Methods

### 4.1. Materials

Gel (Type A, from porcine skin, –NH_2_ content: 4.45 × 10^−4^ mol/g) was obtained from Sigma–Aldrich (Shanghai, China). Dex (M_w_ = 70 kDa), sodium periodate (NaIO_4_, 99.5%), methacrylic anhydride (MA, 96%), phosphate-buffered saline (PBS, pH 7.4), and lithium phenyl–2,4,6–trimethylbenzoylphosphinate (LAP, 98.0%) were purchased from Macklin (Shanghai, China). Cefazolin sodium (CEF, 99%) was gently supplied by Shandong Tianming Pharmaceutical Co., Ltd. (Jinan, China). Dialysis bags with molecular weight cut-offs (MWCO) of 3.5 and 10 kDa were provided by Aladdin (Shanghai, China). Diethylene glycol (DEG, 99%) and ninhydrin (NH, 99%) were purchased from J&K Scientific Ltd. (Beijing, China). The Calcein AM/PI staining kit was sourced from Merck (Darmstadt, Hessen, Germany).

### 4.2. Synthesis of GelMA

**Figure 13:**
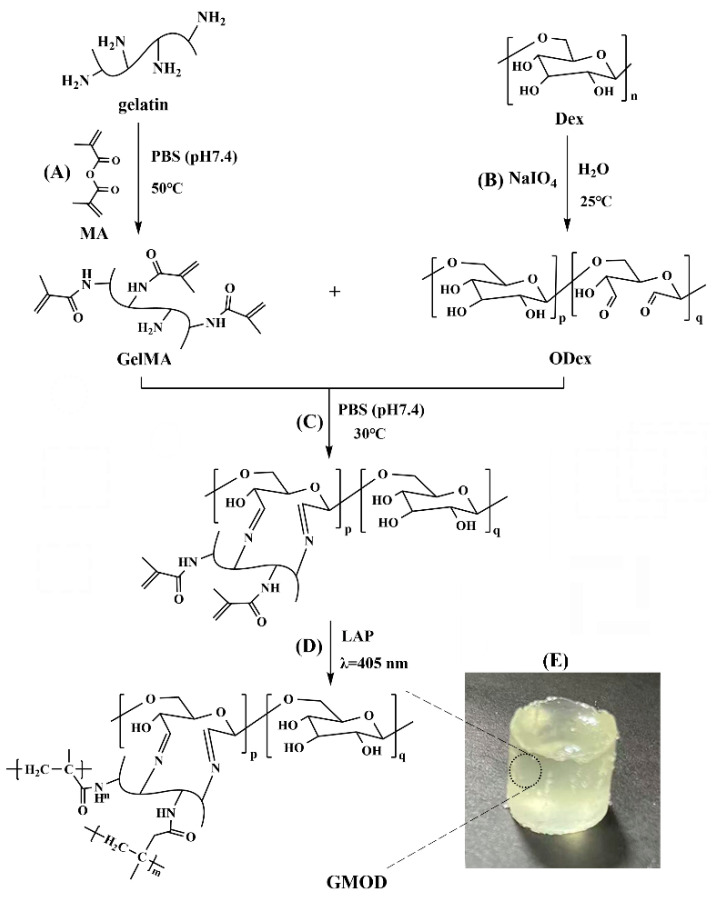
Synthetic routes of (**A**) GelMA and (**B**) ODex; structural schematic diagrams of (**C**) single-crosslinked pre-hydrogels and (**D**) dual-crosslinked GMODs; (**E**) representative GMOD hydrogel.

GelMA was synthesized according to a reported method with minor modifications [51], and the reaction scheme is depicted in Figure 13A. In brief, Gel (10.0 g) was dissolved in PBS (100 mL) under stirring at 50 °C, followed by the dropwise addition of MA (10.0 g, 65 mmol) at a rate of 1 drop/s. The reaction proceeded at 50 °C for 2.5 h and was subsequently quenched with PBS (400 mL) at room temperature. The reaction mixture was transferred into a dialysis bag (MWCO: 10 kDa) and dialyzed against deionized water for 7 days, with the external medium refreshed every 12 h. After that, the solution was centrifuged (3000 rpm, 15 min) to remove insoluble residues, and the supernatant was lyophilized to obtain a white, porous GelMA with a yield of 81–85%.

The chemical structure of GelMA was confirmed by ^1^H NMR (Figure S3). Based on the integration of characteristic proton resonances in the ^1^H NMR spectrum [52] and quantification by the TNBS colorimetric assay [53] (detailed procedures are provided in the Supplementary Materials), the degree of methacrylation (i.e., acylation of –NH_2_ groups) was determined to be approximately 70%.

### 4.3. Synthesis of ODex

Dex (10.0 g) was completely dissolved in deionized water (100 mL) under continuous stirring, followed by the addition of NaIO_4_ (8.1 g, 38 mmol). The reaction was carried out under light-shielded conditions at ambient temperature for 4 h. Subsequently, DEG (4.0 g, 38 mmol) was introduced to terminate the reaction. The resulting solution was transferred into a dialysis bag with a MWCO of 3.5 kDa and dialyzed against deionized water for 3 days, with the external water replaced every 12 h. The dialyzed solution was lyophilized to give a sponge-like ODex with a yield of 75–82%. The synthetic route is illustrated in Figure 13B.

The proton signal at δ 9.79 ppm attributable to –CHO in ODex was confirmed by ^1^H NMR (Figure S4) [54]. The –CHO content in ODex was quantified as 4.1 × 10^−3^ mol/g using the hydroxylamine hydrochloride titration method [55] (detailed procedures are provided in Supplementary Materials).

### 4.4. Preparation of GMOD Hydrogels

GelMA and ODex were individually dissolved in PBS containing LAP (2.5 × 10^−3^ g/mL) at 30 °C with the solid content of 10%. The solutions were first degassed under reduced pressure and then rapidly mixed at predetermined ratios. After gentle shaking to obtain a homogeneous solution, the mixture was incubated in a biochemical incubator at 37 °C for approximately 5 min to form viscous single-crosslinked pre-hydrogels (Figure 13C). Subsequently, the pre–hydrogels were exposed to blue light (λ = 405 nm) to yield GMOD hydrogels with dual-crosslinked network (Figure 13D). A representative hydrogel sample is shown in Figure 13E.

The molar ratios of –NH_2_ in GelMA to –CHO in ODex were set to 10:0, 10:3, 10:5, and 10:7, and the prepared GMODs were designated GMOD−0, −1, −2, and −3, respectively. The corresponding lyophilized samples, obtained by pre-freezing at −50 °C for 8 h followed by freeze-drying under vacuum (2–5 Pa) for 24 h, were denoted as DGMOD−0, −1, −2, and −3.

### 4.5. Instruments and Characterization

#### 4.5.1. Structural Characterization

^1^H NMR spectra were recorded on a Bruker Avance II 400 MHz NMR spectrometer (Rheinstetten, Germany) using D_2_O as the solvent and TMS as the internal standard at room temperature. FT–IR spectra were recorded on an Alpha FT-IR spectrometer equipped with an ATR accessory (Rheinstetten, Germany). Each spectrum was collected in the range of 4000–400 cm^−1^ with a resolution of 4 cm^−1^.

#### 4.5.2. Microstructure

The freeze-dried hydrogels were cryo-fractured in liquid nitrogen, sputter-coated with gold, and subsequently observed under a SU–8010 SEM (Hitachi, Tokyo, Japan).

#### 4.5.3. Thermal Properties

Thermal stability were analyzed by TGA analysis (Q50, TA Instruments, New Castle, DE, USA), which was performed under N_2_ with a heating rate of 15 °C/min over 40–600 °C. Thermal transition was measured by DSC (DSC25, TA Instruments, New Castle, DE, USA), in which samples were first rapidly heated to erase thermal history, followed by a second heating at a rate of 10 °C/min from –50 to 100 °C under N_2_. The thermograms from the second heating run were recorded.

#### 4.5.4. Surface Water Contact Angle

The water contact angle on the hydrogel surface was determined using the sessile drop method with a CAM200 goniometer (KSV, Helsinki, Finland). Prior to measurement, the samples were immersed in deionized water for 30 min, and the excess surface liquid was carefully removed. A 2 μL droplet of deionized water was then deposited onto the hydrogel surface, and an image was captured after 3 s.

#### 4.5.5. Swelling Behavior

The cylindrical hydrogel sample (height: 12 mm; diameter: 30 mm) was weighed (W_0_) and immersed in deionized water at room temperature. At predetermined time intervals, the sample was removed and the surface water was gently blotted with filter paper before weighing (Wₜ). This procedure was repeated until no further weight change. The swelling ratio (SR) was calculated according to the following equation: SR (%) = (W_t_ − W_0_)/W_0_ × 100.

#### 4.5.6. Water-Retention Capacity

The water retention capacity of the prepared hydrogel was evaluated by monitoring time–dependent water loss of the samples. A rectangular freeze-dried hydrogel (50 × 25 × 5 mm^3^) was weighed as *M*_0_ and immersed in deionized water until water absorption equilibrium was reached. The equilibrium-swollen hydrogel with a mass of *M*ₚ were subsequently placed in a biological incubator maintained at 25 °C and 30% RH. At 12 h intervals, the sample was weighed as *M*ₜ. The water loss ratio (WLA) was calculated using the following equation: WLA (%) = [(*M*ₜ − *M*_0_)/(*M*_p_ − *M*_0_)] × 100. A commercial polyurethane (PU) sponge with the same dimensions was used as a comparative sample.

#### 4.5.7. Porosity

The porosity of the freeze-dried hydrogel was determined by the solvent replacement method. Ethanol (*V*_1_) was added into a graduated cylinder, and the DGMOD sample was immersed in ethanol. After the sample reached full swelling, the total volume was recorded as *V*_2_. The swollen sample was then removed, and the residual ethanol volume was recorded as *V*_3_. The porosity was calculated as Porosity% = [(*V*_1_ − *V*_3_)/(*V*_2_ − *V*_3_)] × 100.

#### 4.5.8. Degradability

The degradation behavior of the hydrogel was evaluated by a mass-loss method. An equilibrium-swollen hydrogel sample (*G*_1_) was wrapped in a pre–moistened gauze bag (*G*_0_) and immersed in PBS solution. The sealed sample was incubated in a biological incubator maintained at 37 °C and 50% RH. After removal at predetermined time intervals, the gauze bag was weighed (*G*ₙ) after the excess surface moisture had ceased dripping. The degradation loss rate (DLR) was calculated using the following equation: DLR (%) = (*G*_1_ + *G*_0_ − *G*_t_)/*G*_1_ × 100.

#### 4.5.9. Compression Test

Compression tests of hydrogels were performed on a CT3 texture analyzer (Brookfield, Middleboro, MA, USA) equipped with a circular TA10A probe. Cylindrical hydrogel samples (diameter: 12.0 mm; height: 12.0 mm) were compressed at a compressive rate of 0.01 mm/s up to a maximum strain of 90%.

#### 4.5.10. Drug–Release Behavior

CEF, as a model drug, was first dissolved in deionized water at a concentration of 0.1 mg/mL. Drug-loaded hydrogels were then fabricated using the same procedure as that for the pure hydrogels. The cumulative release profile of CEF from the hydrogels at body temperature over time was determined.

#### 4.5.11. Cytocompatibility Evaluation

The cytotoxicity of the hydrogels was evaluated by the MTT assay according to GB/T 16886.1–2022 using mouse fibroblast cells (L929) [45]. All samples were sterilized by UV irradiation prior to testing. After culturing with the extracts for 72 h, the cells were stained with Calcein AM/PI and the live/dead status were observed with an inverted fluorescence microscope (XPS−15C, RATTOP, Guangzhou, China).

#### 4.5.12. Hemolysis

The hemolysis tests of the hydrogels were performed according to a published method [43].

#### 4.5.13. Antibacterial Properties

The antibacterial activities were performed via inhibition zone method against *E. coli* (GN bacteria) and *S. aureus* (GP bacteria). A bacterial suspension (10^5^ CFU/mL) in a dish was prepared, after which a hydrogel disc (5.0 mm) was placed at the center of the dish. Following incubation at 37 °C for 24 h, the inhibition zone was photographed using a digital camera, and the diameter was measured to quantify antibacterial efficacy.

Detailed experimental procedures for CEF release, cytotoxicity, and hemolysis are provided in the Supplementary Materials.

## Data Availability

The original contributions presented in this study are included in the article/Supplementary Material. Further inquiries can be directed to the corresponding author.

## Supplementary Materials

The following Supplementary Materials can be downloaded at: https://www.mdpi.com/article/10.3390/gels11110871/s1, Figure S1: Formation process of GMOD−2 hydrogels; Figure S2: Images of pristine (left) and swelled (right) GMOD−2; Figure S3: 1H NMR spectra of Gel and GelMA; Figure S4: 1H NMR spectra of Dex and ODex; Table S1: Characteristic values of DGMODs from TGA and DTGA curves; Table S2: Characteristic values of GelMA, ODex and DGMODs from DSC curves.
