# Supplementary material for: Dual-Crosslinked Gelatin/Dextran Medical Hydrogels Based on Aldimine Condensation and Photopolymerization
Source: Gels. 2025 Oct 31;11(11):871. doi: 10.3390/gels11110871 (PMC12652792; doi:10.3390/gels11110871)
Supplement: Supplementary file 1 [file gels-11-00871-s001.zip › gels-3946720-supplementary.pdf]

Supplementary Materials

Dual-Crosslinked Gelatin/Dextran Medical Hydrogels Based on Aldimine Condensation and Photopolymerization

Xia Ding[1], Bing Yang[1], Lei Ni[1], Guangliang Niu[2], Xinyi Si[2], Ning Lu[2], Zhaosheng Hou[2,*]

[1] School of Intelligence Engineering, Shandong Management University, Jinan 250357, China;
[2] College of Chemistry, Chemical Engineering and Materials Science, Shandong Normal University, Jinan 250014, China;
[*] Correspondence: houzs@sdnu.edu.cn

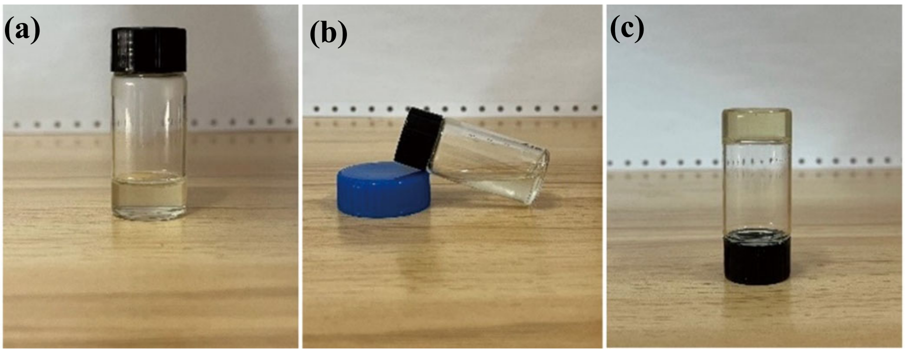

Figure S1. Formation process of GMOD−2 hydrogels.
(a) Freshly mixed solution; (b) primary network formation; (c) secondary network formation.

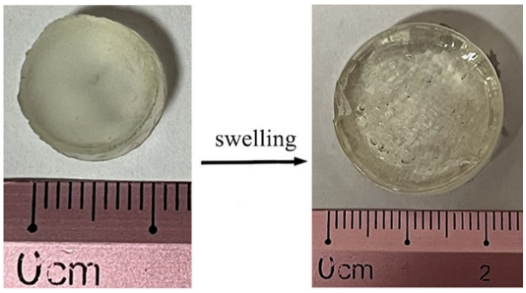

Figure S2. Images of pristine (left) and swelled (right) GMOD−2.

Table S1. Characteristic values of DGMODs from TGA and DTGA curves.

Samples	$T_{5\%}$/℃	T_1/℃	T_2/℃	W_r/%
DGMOD−0	125.3	135.3	264.7	23.1
DGMOD−1	135.2	142.3	290.4	25.1
DGMOD−2	181.1	185.5	309.7	28.0
DGMOD−3	186.2	209.3	333.6	31.2

Table S2. Characteristic values of GelMA, ODex and DGMODs from DSC curves.

Samples	T_m /℃	ΔH /J·g^{-1}
GelMA	82.29	21.3
ODex	93.60	19.1
DGMOD−0	82.75	17.6
DGMOD−1	86.78	14.0
DGMOD−2	90.33	11.7
DGMOD−3	93.78	8.9

1. GelMA

1.1. ^{1}H NMR spectrum of GelMA

GelMA was synthesized via condensation reaction between Gel and MA. The chemical structure of GelMA was characterized by NMR technique, and the ^{1}H NMR spectra of Gel and GelMA are presented in Figure S1. Compared with the ^{1}H NMR spectrum of Gel (Figure S3a), new proton signals at δ 1.92 ppm and at δ 5.41, 5.65 ppm appeared in the ^{1}H NMR spectrum of GelMA (Figure S3b), which were assigned to the -CH_3 protons and −CH_2=C− protons of the methacryloyl groups, respectively. In addition, a notable reduction in the proton peak area at δ 2.98 ppm is observed, corresponding to the methylene protons (ε-CH_2) adjacent to −NH_2 in the lysine units of Gel. These spectral changes indicated the successful acylation reaction between MA and the −NH_2 of Gel, resulting in the formation of GelMA.

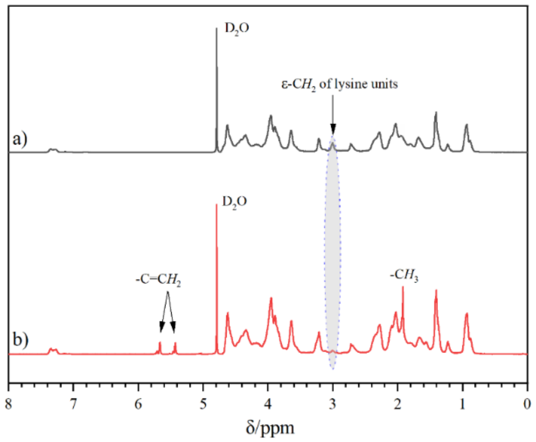

Figure S3. ^{1}H NMR spectra of (a) Gel and (b) GelMA.

1.2. Degree of methacrylation of Gel

The degree of methacrylation (DM) was defined as the percentage of -NH_2 in Gel that were acylated in GelMA. It was determined by the following two methods: ^{1}H NMR [52] and TNBS colorimetry [53].

^{1}H NMR: The DM in GelMA was determined through the change in integration area at δ 2.98 ppm. According to the following equation, the DM_{NMR} was calculated to be 70.2%,

$$DM_{NMR}(\%) = \frac{I_0 - I_g}{I_g} \times 100$$

where I_0 and I_g were integral area of the peaks at δ 2.98 ppm for Gel and GelMA.

TNBS colorimetry: The DM was quantitatively determined by measuring the residual free −NH_2 groups using the 2,4,6-trinitrobenzene sulfonic acid (TNBS, Sigma-Aldrich, Shanghai, China) colorimetry. GelMA and Gel were individually dissolved in 0.1 mol/L $NaHCO_3$ buffer to obtain 1.6 mg/mL solutions. An 0.5 mL of each solution was mixed with 0.25 mL of 0.01% TNBS solution, followed by incubation at 37 °C for 2 h. Subsequently, the reaction was quenched by adding 500 μL of 10% (w/v) sodium dodecyl sulfate and 250 μL of 0.1 mol/L HCl. The absorbance of each sample was recorded at λ = 335 nm, and the DM was calculated to be

69.8% according to the following equation:

$$DM_{TNBS}(\%) = [1 - \left(\frac{A_{GelMA}-A_c}{A_{Gel}-A_c}\right)] \times 100$$

where the A_{GelMA}, A_{Gel}, and A_c are the absorbance of GelMA, Gel, and control, respectively.

2. ODex

2.1. ^{1}H NMR spectrum of ODex

Native dextran was oxidized by $NaIO_4$ to produce ODex with multiple -CHO groups. the chemical structure of Dex and ODex was characterized by ^{1}H NMR technique, as illustrated in Figure S2. Compared with the ^{1}H NMR spectrum of Dex (Figure S4a), new proton signal at δ 9.79 ppm was observed in the ^{1}H NMR spectrum of ODex (Figure S4b), which were ascribed to the characteristic signal of -CHO groups. The emergence of the characteristic signal served as definitive evidence for the conversion of Dex to -CHO functionalized ODex. However, quantification of the -CHO content based on NMR spectrum was not feasible due to limitations in signal resolution.

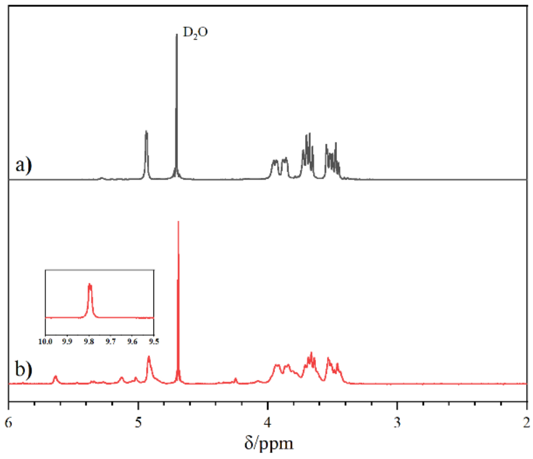

Figure S4. ^{1}H NMR spectra of (a) Dex and (b) ODex.

2.2. -CHO content in ODex

The -CHO content of ODex was quantified by the hydroxylamine hydrochloride method [55]. Briefly, 2.0 g of hydroxylamine hydrochloride was dissolved in an appropriate amount of deionized water, followed by the sequential addition of methyl orange indicator and 2.0 g of the ODex sample into the solution. The mixture was allowed to react under continuous stirring at ambient temperature for 2 h. Subsequently, the residual hydroxylamine was titrated with a standardized NaOH solution (1.0 mol/L) until the color of the system shifted from the initial red to yellow. The consumed volume of NaOH was recorded, and the -CHO content (C_{-CHO}) was determined to be 4.1 mmol/g according to following equation:

$$C_{-CHO}(\text{mmol/g}) = \frac{(V_2 - V_1)C_{NaOH}}{m}$$

where V_1 (L) and V_2 (L) are the volume of the NaOH solution consumed in the blank and sample titration, respectively; C_{NaOH} is the concentration of NaOH solution (1.0 mol/L); m (g) is the mass

of ODex sample.

3. Instruments and Characterization

3.1. Drug–Release Behavior

The drug-loading hydrogel disc (diameter: 6 mm; height: 3 mm) containing approximately 10 mg of CEF were immersed in 10 mL of PBS and incubated at 37 °C. At predetermined intervals, 1 mL of the release medium was withdrawn and replaced with an equal volume of fresh PBS. The absorbance of the collected supernatant was recorded at 272 nm using UV–visible spectrophotometry. The released drug concentration was determined from a standard calibration curve, and the cumulative drug release (CDR) was calculated according to following equation:

$$CDR(\%) = \frac{c_n v_n v_{10}}{v_1 \left(m_0 - \frac{c_{n-1} \times v_{n-1} \times v_{10}}{v_1} \right)} \times 100$$

where c_n and v_n denote the concentration and solvent volume at the nth sampling point, v_{10} is the total PBS volume (10 mL), and v_1 represents the withdrawn volume.

3.2. Cytotoxicity

The hydrogel samples were immersed in 2.5 mL of DMEM supplemented with 10% PBS and incubated for 24 h at 37 ± 0.5 °C to obtain the extracts. The supernatant was then filtered through a 0.22 μm membrane and subsequently diluted with an equal volume of fresh medium. An aliquot (100 μL) of the diluted extract was added to each well of a 24-well plate seeded with L929 fibroblast cells (~1 × 10^5 cells/well). Cells cultured in untreated medium served as the control group. After incubation for 72 h at 37 °C in 5% CO_2, cell viability was assessed via the MTT assay. The optical density (OD) was recorded at 570 nm using a microplate reader, and the cell survival rate (CSR) was calculated using the following equation:

$$CSR(\%) = \frac{A_s - A_b}{A_c - A_b} \times 100$$

where A_s, A_b, and A_c represent the absorbance values of the sample, blank, and control groups, respectively.

3.3. Hemolysis

Blood was obtained from the hearts of healthy rabbits, and 5% sodium citrate was added as an anticoagulant. The anticoagulated blood (8.0 mL) was then diluted with 10.0 mL of normal saline. A tube containing the hydrogel (2.0 g) dispersed in 10 mL of normal saline was incubated at 37 °C for 0.5 h, after which 0.2 mL of the diluted blood was introduced and the mixture was further incubated for 1 h. Following centrifugation for 5 min, the absorbance of the supernatant was measured at 540 nm using an enzyme-linked immunosorbent assay reader. normal saline and distilled water without hydrogel served as the negative and positive controls, respectively. The hemolysis ratio (HR) was calculated according to the following equation:

$$\text{HR}(\%) = \frac{A_o - A_{nc}}{A_{pc} - A_{nc}} \times 100$$

where A_o, A_{nc}, and A_{pc} represent the absorbance values of the sample, negative control, and positive control, respectively.

References

52. Hoch, E.; Schuh, C.; Hirth, T.; Tovar, G.E.M.; Borchers, K. Stiff gelatin hydrogels can be photo-chemically synthesized from low viscous gelatin solutions using molecularly functionalized gelatin with

53. a high degree of methacrylation. *J. Mater. Sci. Mater. Med.* **2012**, *23*, 2607–2617.

Shirahama, H.; Lee, B.H.; Tan, L.P.; Cho, N.J. Precise tuning of facile one-pot gelatin methacryloyl (GelMA) synthesis. *Sci. Rep.* **2016**, *6*, 31036.

55. Zhao, H.; Heindel, N.D. Determination of degree of substitution of formyl groups in polyaldehyde dextran by the hydroxylamine hydrochloride method. *Pharm. Res.* **1991**, *8*, 400–402.
